# Impact of *Ae-GRD* on Ivermectin Resistance and Its Regulation by miR-71-5p in *Aedes aegypti*

**DOI:** 10.3390/insects15060453

**Published:** 2024-06-14

**Authors:** Lingling Yu, Yanan Yin, Qiuhui Wang, Peizhen Zhao, Qian Han, Chenghong Liao

**Affiliations:** 1 Laboratory of Tropical Veterinary Medicine and Vector Biology, School of Life and Health Sciences, Hainan University, Haikou 570228, China; gzzljszyyx@163.com (L.Y.); yinyanan@hainanu.edu.cn (Y.Y.); 19359281004@163.com (Q.W.); peizhen.zhao@hainanu.edu.cn (P.Z.); 2 Hainan One Health Key Laboratory, Hainan University, Haikou 570228, China; 3 Hainan International One Health Institute, Hainan University, Haikou 570228, China

**Keywords:** *Aedes aegypti*, iGABAaR, *Ae-GRD*, RNA interference, microRNA, ivermectin

## Abstract

**Simple Summary:**

Ivermectin (IVM), a macrolide insecticide, targets the ionotropic gamma-aminobutyric acid receptor (iGABAR) and plays a crucial role in controlling *Aedes aegypti* mosquitoes and researching mosquito drug resistance. This study marks the first characterization of the GRD subunit (*Ae-GRD*) of the *Ae. aegypti* iGABAR. We discovered that the expression of *Ae-GRD* is negatively regulated by miR-71-5p, a finding supported by both in vitro cell studies and in vivo microinjection experiments. Using RNA interference (RNAi) techniques and bioassays, we found that silencing *Ae-GRD* through dsRNA microinjection decreased the susceptibility of *Ae. aegypti* to IVM. Additionally, similar regulation of *Ae-GRD* by miR-71-5p altered the sensitivity of the mosquitoes to the insecticide.

**Abstract:**

iGABAR, a member of the Cys-loop ligand-gated ion channel superfamily, is a significant target of the insecticide ivermectin (IVM). GRD is the potential subunit of the insect iGABAR. However, little information about GRD in *Ae. aegypti* has been reported. In this study, we involved cloning and characterizing the iGABAR subunit *GRD* of *Ae. aegypti* (*Ae-GRD*). Sequence analysis indicated that *Ae-GRD*, as part of the cysteine-loop ligand-gated ion channel family, is similar to other insect *GRD*. RNA interference (RNAi) was employed to explore IVM resistance in *Ae. aegypti*, resulting in a significant reduction in *Ae-GRD* expression (*p* < 0.05), and the mortality of *Ae. aegypti* adults with *Ae-GRD* knockdown was significantly decreased after exposure to ivermectin. Bioinformatics prediction identified miR-71-5p as a potential regulator of *Ae-GRD*. In vitro, dual-luciferase reporter assays confirmed that *Ae-GRD* expression was regulated by miR-71-5p. Microinjection of miR-71-5p mimics upregulated miR-71-5p expression and downregulated *Ae-GRD* gene expression, reducing mortality by 34.52% following IVM treatment. Conversely, microinjection of a miR-71-5p inhibitor decreased miR-71-5p expression but did not affect the susceptibility to IVM despite increased *Ae-GRD* expression (*p* < 0.05). In conclusion, *Ae-GRD*, as one of the iGABA receptor subunits, is a potential target of ivermectin. It may influence ivermectin resistance by modulating the GABA signaling pathway. The inhibition of *Ae-GRD* expression by miR-71-5p decreased ivermectin resistance and consequently lowered the mortality rate of *Ae. aegypti* mosquitoes. This finding provides empirical evidence of the relationship between *Ae-GRD* and its miRNA in modulating insecticide resistance, offering novel perspectives for mosquito control strategies.

## 1. Introduction

*Aedes aegypti* (*Ae. aegypti*) is a principal vector for arthropod-borne diseases [1], posing significant threats to human health through the pathogen transmission activities of its female adults, which feed on blood before and after mating [2]. With no effective vaccines or treatments currently available for many of these diseases, control strategies primarily focus on vector management [1]. Chemical insecticides are central to mosquito control efforts [3]. Unlike mammals, insects have a reduced capacity for detoxification, allowing insecticides to persistently target neuronal pathways and exert prolonged neurotoxic effects [4]. Neurotoxic insecticides, for instance, swiftly incapacitate or kill pests by targeting multiple neural sites, categorized into four main groups: acetylcholinesterase (aChE), nicotinic acetylcholine receptors (NaChRs), gamma-aminobutyric acid receptors (GABARs), and voltage-gated sodium channels (VGSCs) [4].

**Figure 1:**
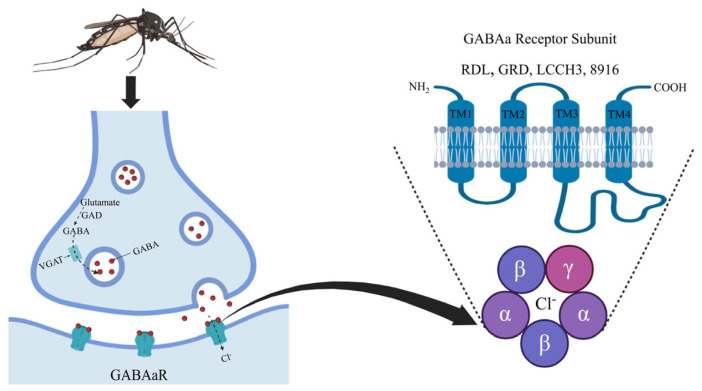
Diagram of GABA signaling pathway and GABAaR subunits in the mosquito.

GABA, an inhibitory neurotransmitter [5], predominantly mediates rapid inhibitory neurotransmission and acts as an excitatory mediator during critical neuronal developmental phases or under pathological conditions [6,7]. GABARs are part of the cysteine loop ligand-gated ion channel superfamily (cys-loop LGICs) [8]. Insect ionotropic GABARs (iGABARs) comprise a-type GABARs (GABAaR), c-type GABARs (GABAcR), and the metabotropic b-type GABARs (GABAbR) [4]. This study focuses primarily on the GABAaR, referred to hereafter as iGABAaR unless otherwise specified. The iGABAaR is mainly composed of the N-terminal extracellular structural domain, four transmembrane structural domains (M1–M4) consisting of α-helices, the “cys-loop” between the N-terminal and transmembrane structural domains, and the C-terminal extracellular structural domain. The binding of GABA to the iGABAaR opens the pentameric ion channel and allows chloride ions to flow inward, leading to hyperpolarization of the membrane potential (Figure 1).

In insects, iGABAaR subunits primarily comprise RDL, GRD, LCCH3, and 8916 (Figure 1). Research has identified RDL as the primary binding site for insecticides [9]. In *Ae. aegypti*, RDL was targeted by ivermectin and florellan [10]. When expressed in *Xenopus laevis* oocytes, RDL formed pentameric anion-selective channels. Conversely, GRD and LCCH3 alone did not form functional ion channels; however, their co-expression resulted in heteropentameric cation-selective channels in *Drosophila melanogaster*, *Apis mellifera*, and *Pediculus humanus* [11,12,13]. RDL also functioned as an anion channel when co-expressed with either GRD or LCCH3 [11,12,13]. Furthermore, in *Chilo suppressalis*, 8916 interacted with LCCH3 to form cation-selective channels sensitive to various insecticides [14]. These findings suggest that additional iGABAaR subunits could also serve as potential targets for insecticides [14].

iGABARs are critical targets for various insecticides [15], such as cyclopentadienes (e.g., dieldrin), phenylpyrazoles (e.g., fipronil), and macrolides (e.g., ivermectin) [4,15]. Mutations in iGABAR are a leading cause of insecticide resistance among insects, underscoring the need for ongoing research into their modulation and resistance mechanisms. The complexity of insecticide interactions with these receptors is further exemplified by the mode of action of ivermectin (IVM), a derivative of the macrolide insecticide abamectin (AVM), exhibiting higher activity compared with its abamectin [16]. This class of insecticides disrupts normal GABA signaling by activating voltage-gated chloride ion (Cl^−^) channels, resulting in the influx of Cl^−^ ions. This influx causes hyperpolarization of the nerve membrane potential, placing the nerve membrane in an inhibitory state and blocking nerve impulse conduction, ultimately leading to insect death. The insecticide represented by abamectin has been found to act on almost all chloride channels, such as γ-aminobutyric acid-gated chloride channels [17], glutamate-gated chloride channels [18], and glycine ligand-gated chloride channels [19]. In *Ae. aegypti*, IVM not only targets the iGABAaR subunit RDL for insecticidal action [10] but also impacts egg production and hatching rates following treatment with various concentrations of IVM.

MicroRNA is a noncoding single-stranded RNA, typically comprising 18–25 nucleotides [20], and most mosquito miRNAs are highly conserved in sequence [21]. A single mRNA may contain multiple binding sites for the same or different miRNAs, allowing several miRNAs to cooperatively repress gene expression. Consequently, differential miRNA expression is frequently observed in studies related to mosquito growth, development, reproduction, and insecticide resistance [22]. In *Culex pipiens pallens*, numerous miRNAs displayed differential expression patterns before and after deltamethrin exposure [23]. Notably, the downregulation of miR-71 was associated with increased mortality in deltamethrin-resistant mosquitoes [23], while miR-279-3p was implicated in deltamethrin resistance in *Cx. pipiens pallens* by targeting the CYP325BB1 gene [24].

In *Ae. aegypti*, only *Ae-RDL* and *Ae-LCCH3* have been identified [25], while *Ae-GRD* and *Ae-8916* remain unvalidated. Despite evidence supporting the involvement of miRNAs in the development of insecticide resistance in mosquitoes, there remains a significant research gap concerning their role in *Ae. aegypti*. The specific targeting mechanisms of miRNAs and their potential involvement in developing resistance to particular insecticides in *Ae. aegypti* are yet to be fully explored.

This study aims to investigate the influence of the *Ae-GRD* subunit of the iGABAaR in *Ae. aegypti* on the resistance mechanisms against the insecticide ivermectin (IVM) and to elucidate the regulatory roles of miRNAs in this process. Given the critical role of GABAergic neurotransmission in insecticide targeting and the emerging evidence of miRNA involvement in gene regulation under insecticidal stress, understanding these interactions at the molecular level could provide new avenues for controlling resistance in mosquito populations. Utilizing comprehensive transcriptomic and genomic data, this research focuses on the bioinformatics analysis of the full-length sequence of Ae-GRD and investigates its functional implications in IVM resistance through advanced molecular techniques such as RNA interference (RNAi) and dual-luciferase reporter assays. By exploring both genetic and post-transcriptional modifications influencing *Ae-GRD*, this study seeks to reveal novel insights into the mosquito’s adaptive responses to insecticides, potentially guiding the development of more effective mosquito control strategies.

## 2. Materials and Methods

### 2.1. Mosquito Breeding

*Ae. aegypti* (the Rockefeller strain, provided by the Beijing Institute of Microbiology and Epidemiology, Beijing, China) eggs, maintained by our laboratory, were incubated under controlled environmental conditions at a temperature of 25 ± 2 °C, humidity of 80 ± 5%, and a light/dark cycle of 12 h/12 h. Larvae were reared in dechlorinated ionized water and fed with mouse chow of specific pathogen-free (SPF) grade. Adults were housed in mosquito cages and sustained on 8% sucrose solution. Female adult *Ae. aegypti*, reared for two days post fledging, were selected for inclusion in the experiments.

### 2.2. Identification of Ae-GRD

*GRD* nucleotide sequences from various insects were retrieved from the National Center for Biotechnology Information (NCBI) database and analyzed for homology. Primers were designed based on sequences with high homology. Total RNA was extracted from *Ae. aegypti* using the Trizol kit (Sangon Biotech, Shanghai, China), and RNA integrity was assessed via 1% gel electrophoresis; RNA concentration was measured with a micro-nucleic acid detector (Aoyi Instruments, Shanghai, China). Reverse transcription was performed on the total RNA using the SPARKscript II RT Plus kit (SparkJade, Shandong, China) to synthesize first-strand cDNA. This cDNA served as the template for PCR amplification of the *Ae-GRD* target fragment. The PCR reaction mixture included 12.5 μL of 5 High-Fidelity 2 × master mix, 1.25 μL of forward primer, 1.25 μL of reverse primer, 1 μL of cDNA, and 9 μL of ddH_2_O. The PCR program consisted of an initial denaturation at 98 °C for 2 min, followed by 30 cycles of 98 °C for 10 s, 52 °C for 30 s, and 72 °C for 45 s, with a final extension at 72 °C for 10 min. PCR products were purified using a kit from Sangon Biotech, Shanghai, China. The purified *Ae-GRD* fragment was cloned into the pMD-18T vector (Takara, Beijing, China) and transformed into DH5α *Escherichia coli* (*E. coli*) competent cells (Weidi, Shanghai, China). Plasmids extracted from the DH5α *E. coli* cells using the Plasmid Extraction Kit (Sangon Biotech, Shanghai, China) were sequenced to confirm their sequences.

### 2.3. Bioinformatics Analysis and Potential miRNAs Prediction of Ae-GRD

As described previously, the *Ae-GRD* sequence obtained by cloning was subjected to a BLAST (Basic Local Alignment Search Tool) comparison with the *Ae. aegypti* genome sequence (NC_035107.1) available in the NCBI database, resulting in the acquisition of the full-length *Ae-GRD* cDNA (LOC5566204). Amino acid sequences of GRD from various insects were also retrieved from the NCBI database. These sequences were compared using DNAMAN 9.0 software, while transmembrane structural domains were analyzed using TMHMM 2.0 software. A phylogenetic tree was constructed by the neighbor-joining method [26,27] using MEGA11 [28,29]. To identify potential miRNAs that might regulate *Ae-GRD*, the 3’ UTR sequence of *Ae-GRD* was obtained from the NCBI database. Potential regulatory miRNAs were predicted using three online tools: TargetScan (https://www.targetscan.org/vert_80/, accessed on 16 April 2023), RNAhybrid (https://bibiserv.cebitec.uni-bielefeld.de/rnahybrid, accessed on 23 April 2023), and miRanda (https://www.miranda.software/, accessed on 8 April 2023). Predictions from these tools were intersected to identify likely miRNAs targeting the 3’ UTR of *Ae-GRD* in *Ae. aegypti*.

### 2.4. In Vitro Synthesis and Microinjection of dsRNAs

The *E. coli β-glucosidase* gene (GUS) [30] served as a negative control, with DEPC-H_2_O used as a blank control and dsRNA-*Ae-GRD* designated as the experimental group. Primers specific for RNAi were designed for the coding region (CDS) of *Ae-GRD* using Primer 5.0, incorporating the T7 promoter sequence (taatacgactcactataggg) at the 5’ end. Using *Ae. aegypti* cDNA as a template, PCR was performed to amplify the target fragment, which was then purified. The purified PCR fragments underwent in vitro dsRNA synthesis using the T7 RNAi Transcription Kit (Vazyme, Nanjing, China), following the manufacturer’s instructions. The dsRNA purification protocol involved combining 40 μL of dsRNA with 4 μL of 3 M sodium acetate (pH = 5.2) (Macklin, Shanghai, China) and 40 μL of isopropanol (Mreda, Beijing, China), chilling the mixture in an ice bath for 10 min, followed by centrifugation and supernatant removal. The residue was washed with 1 mL of 70% DEPC-treated ethanol, centrifuged again, and the resulting precipitate was resolubilized in DEPC-treated water to yield purified dsRNA.

Quality assessment of the PCR reactions and dsRNA was conducted as described in Section 2.2. Female *Ae. aegypti* mosquitoes, normally fed for 2 days post feathering, were subjected to a 24 h starvation period, then paralyzed by placement in a −20 °C refrigerator for 3 min and subsequently arranged on ice [10,31,32]. Each mosquito was injected at the thoracic–ventral junction using a microsyringe (Eppendorf, Hamburg, Germany) with 1 μL of purified dsRNA solution (2000 ng/μL) [10,33]. Three replicates were set for each group, with 50 mosquitoes injected per replicate, and the experiment was conducted three times. The expression level of *Ae-GRD* was analyzed 24 h post injection using real-time quantitative reverse transcription PCR to assess the RNAi effect. Details of the primers used in dsRNA interference experiments are provided in Table S1.

### 2.5. Dual-Fluorescent Vector Construction

As outlined in Section 2.2, the full-length 3’ noncoding region (UTR) of *Ae-GRD* was amplified by PCR using *Ae. aegypti* cDNA as a template. The amplified gene fragment was initially cloned into the pMD-18T vector and subsequently transferred into DH5α *E. coli*. Following this, the recombinant plasmid was extracted. The extracted plasmid underwent double digestion with *Xho* I and *Sca* I restriction endonucleases (Thermo Fisher Scientific, Pittsburgh, PA, USA). The digested fragments were then purified using the SanPrep Column DNA Gel Extraction Kit (Sangyo, Shanghai, China) and subsequently cloned into the pmirGLO Dual-Luciferase Expression Reporter Vector (Promega, Beijing, China) to create luciferase constructs. The newly formed recombinant plasmids were transformed into DH5α *E. coli* cells, which were cultured in a medium overnight to facilitate plasmid amplification. Plasmid extraction was conducted using the SanPrep Endotoxin-Free Plasmid Mini Kit (Bioengineering, Shanghai, China), followed by sequencing to confirm the accuracy of the constructs.

### 2.6. HEK-293T Cell Line Culture and Dual-Luciferase Activity Assay

HEK293T cells, kindly provided by Mr. Dayong Wang from the School of Pharmaceutical Sciences at Hainan University, were cultured in DMEM medium supplemented with 10% fetal bovine serum (Bio channel, Nanjing, China) and maintained at 37 °C in a 5% CO_2_ atmosphere. For transfection, cells were seeded into 24-well plates and allowed to culture for 24 h. Transfection was carried out using FuGENE 6 transfection reagent (Promega, Madison, WI, USA). Each well received 4 μL of reporter gene recombinant plasmid, 1.25 μL of miRNA mimic or negative control (NC) (GenePharma, Shanghai, China), 2 μL of FuGENE 6 transfection reagent, and 94.75 μL of DMEM medium without fetal bovine serum. The sequences of the mimics and NC are listed in Table S1.

Forty-eight hours post transfection, 100 μL of 1x PLB cell lysate (Promega, Madison, WI, USA) was added to each well to lyse the cells, which were then transferred to 1.5 mL centrifuge tubes. The Dual-Luciferase^®^ Assay System kit (Promega, Madison, WI, USA) was employed to measure luciferase activity. Initially, 100 μL of Luciferase Assay Reagent II was added to each tube to measure firefly luciferase activity, followed by 100 μL of Stop & Glo^®^ Reagent to measure Renilla luciferase activity. The results were normalized to the ratio of firefly luciferase activity/sea kidney firefly luciferase. Each experimental condition was replicated three times in the assay, and the experiment was conducted in triplicate to ensure reproducibility.

### 2.7. Inhibition/Overexpression of miR-71-5p in Ae. aegypti

Overexpression and inhibition of miR-71-5p in *Ae. aegypti* were achieved through microinjection of miR-71-5p mimics and inhibitors, respectively. As detailed in Section 2.2, selected *Ae. aegypti* mosquitoes were paralyzed prior to injection. The miR-71-5p mimics, mimics NC, miR-71-5p inhibitor, and inhibitor NC were injected under uniform conditions. For each treatment group, three replicates were established, with 50 mosquitoes injected per replicate, and the entire experiment was conducted three times to ensure consistency and reliability. Twenty-four hours post injection, the relative expression levels of miR-71-5p and *Ae-GRD* were quantified using real-time quantitative reverse transcription PCR (qRT-PCR). The sequences for the inhibitors and their corresponding negative controls are detailed in Table S1.

### 2.8. Real-Time qRT-PCR Analysis of Ae-GRD and miR-71-5p

Following microinjection in *Ae. aegypti*, total RNA was extracted using the Trizol kit as described in Section 2.2. The relative expression levels of the target genes were assessed by real-time fluorescence qPCR after reverse transcription. For the experimental and control groups injected with dsRNA, 1 μg of total RNA was reverse transcribed to obtain the first strand of cDNA using the SPARKscript II RT Plus Kit (SparkJade, Qingdao, China). For the groups injected with miR-71-5p mimic and inhibitor, 1 μg of total RNA was reverse transcribed using the miRNA 1st Strand cDNA Synthesis Kit (by stem-loop) (Vazyme, Nanjing, China).

Following the manufacturer’s instructions, the synthesized cDNA was used as a template for qPCR employing 2× SYBR Green qPCR Mix (SparkJade, Qingdao, China). The reactions were performed on a LightCycler 96. The qPCR reaction mixture comprised 5 μL of 2 × SYBR Green qPCR Mix, 0.5 μL each of forward and reverse primers, 3 μL of ddH_2_O, and 1 μL of cDNA solution. The qPCR cycling conditions were set as follows: initial denaturation at 95 °C for 10 min, followed by 40 cycles of 95 °C for 10 s, 55 °C for 10 s, and 72 °C for 30 s. Each sample group included three replicates, with three mosquitoes per replicate, and four replicate wells per sample during the setup. RPS17 served as the internal reference gene for *Ae-GRD* expression, while U6 was used for miR-71-5p expression. The cycling thresholds (Ct) of U6 and RPS17 were utilized to normalize the Ct values of miR-71-5p and *Ae-GRD*. Data analysis was conducted using the 2^−ΔΔct^ method. Specific primer sequences used in the qPCR reactions are detailed in Table S1.

### 2.9. Bioassays

Adult mosquitoes were collected from control groups and *Ae-GRD* knockdown groups after microinjection. Thirty individuals were collected from each group (*n* = 3) and placed in mosquito cages for subsequent dosing experiments. IVM (I811964), sourced from Macklin (Shanghai, China), was dissolved in dimethyl sulfoxide (DMSO, QN0747, Biorebo Technology Co., LTD, Shenzhen, China) [10,34,35] to prepare a 15 mg/mL solution. This solution was then mixed with an 8% sucrose solution, and the mixture contained  <1% organic solvent [35], then 10 mL of the mixture was applied to each sponge block. These sponge blocks were placed within mosquito cages to serve as feed for the injected *Ae. aegypti* mosquitoes. The mortality rate of the mosquitoes was recorded 24 h after they were exposed to the treated sponges.

### 2.10. GABA Content Determination

Twenty-four hours following dsRNA injection, experimental and control groups were sampled. Fifteen *Ae. aegypti* mosquitoes were collected from each group, and 400 μL of 1xPBS (pH = 7.4) was added to each for lysis. Subsequently, the supernatant was collected (5000 rpm, 15 min). The supernatants were then assayed for GABA content using an insect GABA enzyme immunoassay kit (Spbio, Wuhan, China), following the manufacturer’s instructions. This procedure was repeated three times to ensure the reliability of the results.

### 2.11. Statistical Analysis

All experimental data were statistically analyzed and graphed using GraphPad Prism version 6.02 (GraphPad Software, San Diego, CA, USA). A *t*-test was employed for comparisons between two groups, while one-way ANOVA was used for comparisons among more than two groups. A *p*-value of less than 0.05 was considered statistically significant, with significance levels denoted as * *p* < 0.05, ** *p* < 0.01, *** *p* < 0.001, and **** *p* < 0.0001.

## 3. Results

### 3.1. Cloning and Sequence Analysis of Ae-GRD

A 720-base-pair (bp) fragment of *Ae-GRD* was successfully amplified by PCR using insect GRD conserved region primers and *Ae. aegypti* cDNA as the template. Subsequent NCBI sequence analysis identified the full sequence of *Ae-GRD* (LOC5566204). The full-length cDNA of *Ae-GRD* spans 3531 bp, encompassing a 2052 bp open reading frame (ORF) that encodes 492 amino acids, a 682 bp 5’ untranslated region (UTR), and a 781 bp 3’ UTR.

**Figure 2:**
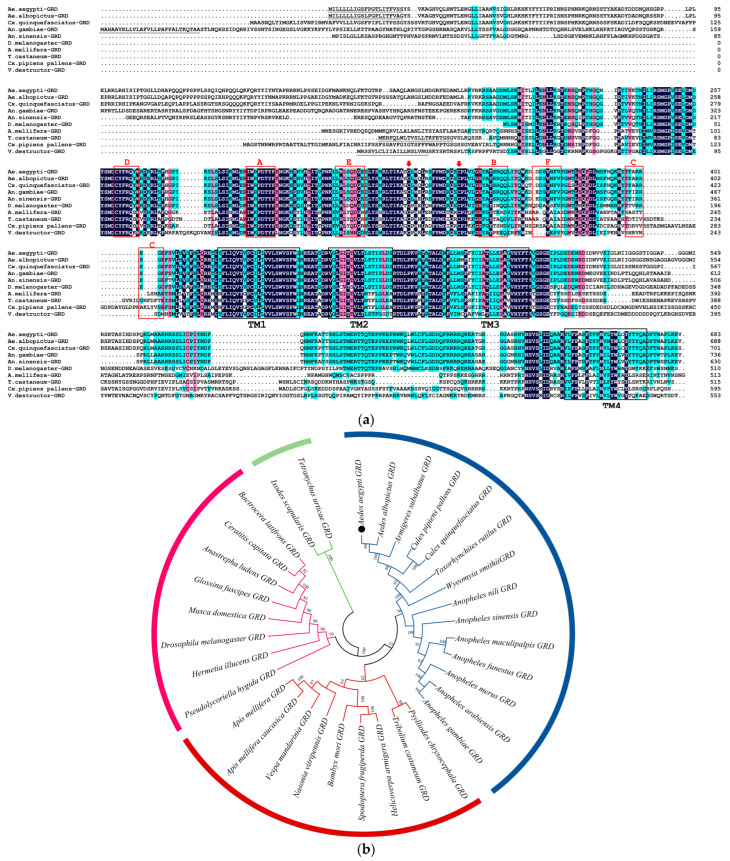
Sequence analysis of Ae-GRD: (**a**) Amino acid sequence comparison and analysis of GRD subunits in *Ae. aegypti* and other insects. Sequence homology is highlighted as dark blue for sequences with ≥100% homology, pink for ≥75% homology, and light blue for ≥50% homology. Key structural features are indicated: red arrows highlight two typical cysteine residues characteristic of the cys-loop LGICs, gray underlining denotes signal peptide regions at the N-terminus, black boxes outline the four transmembrane domains (TM1-TM4), and red boxes identify ligand-binding related structural domains (loops A–F). (**b**) Phylogenetic tree of GRD proteins of *Ae. aegypti* and other insects. The numbers above the branches represent the bootstrap values for each branch (1000 replications). The black dot showed the sequence of Ae-GRD in our study. Taxonomically related organisms are shown by the same color code.

Multiple sequence alignment demonstrated that Ae-GRD shares homologies of 91.73%, 77.98%, 65.45%, 58.08%, 28.83%, 33.10%, 30.47%, 31.59%, and 19.5% with GRDs from *Aedes albopictus, Culex quinquefasciatus, Anopheles sinensis, Anopheles gambiae, Culex pipiens pallens, Varroa destructor, Tribolium castaneum, Apis mellifera,* and *Drosophila melanogaster* in amino acid sequences, respectively. Sequence analysis indicated that Ae-GRD, as part of the cysteine-loop ligand-gated ion channel family, shares common features with other insect GRD subunits. Each subunit contains six N-terminal extracellular loop structures (loops A-F) and four transmembrane regions (TM1-4), with a high degree of structural similarity and conservation of amino acid sequences across these regions (Figure 2a).

GRD sequences of other insect species were downloaded from NCBI. The phylogenetic relationships were analyzed using MEGA11 [29]. All compared GRD protein sequences clustered together among themselves according to their taxonomic rank, and homologs of related organisms showed closer relationships. Evolutionary trends supported their genetic diversity and conserved relationships (Figure 2b). The amino acid numbers of species are shown in Table S2.

### 3.2. RNAi Efficiency of Injected dsRNA-Ae-GRD against Ae. aegypti, with Changes in Ivermectin Susceptibility

**Figure 3:**
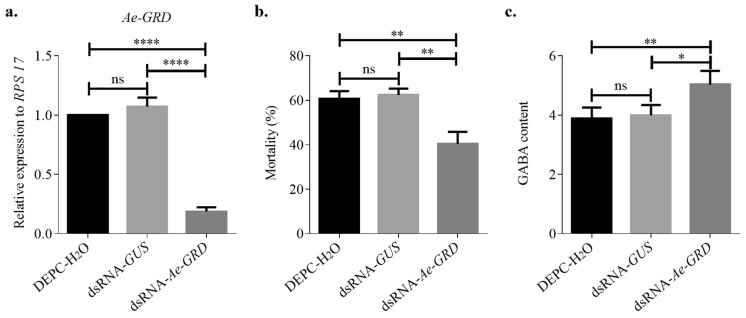
Microinjection of dsRNA for RNAi in *Ae. Aegypti*: (**a**) RNAi significantly reduced the relative expression of *Ae-GRD* mRNA. (**b**) After feeding on a mixture containing 15 mg/mL ivermectin and 8% sucrose solution for 24 h, the mortality rate in the dsRNA-*Ae-GRD* group was 40.37%, which was lower than those observed in the blank and negative control groups. (**c**) GABA content in *Ae. aegypti* was significantly increased following RNAi treatment. Data represent three biological replicates, each consisting of three technical replicates. Statistical analysis was conducted using one-way ANOVA. “ns” indicates no significant difference, while asterisks denote levels of statistical significance: * *p* < 0.05, ** *p* < 0.01, **** *p* < 0.0001.

To elucidate the role of Ae-GRD in IVM resistance in *Ae. aegypti*, in vitro-synthesized and purified dsRNA-*Ae-GRD* was microinjected, with corresponding control groups established. The relative expression levels of *Ae-GRD* post-RNAi were quantified using qPCR. Twenty-four hours following microinjection, there was no significant difference in *Ae-GRD* expression between the dsRNA-*GUS* control group and the DEPC-H_2_O group. However, transcript levels of *Ae-GRD* in the dsRNA-*Ae-GRD* group were significantly reduced by 81.34% compared with the DEPC-H_2_O group and by 82.59% compared with the dsRNA-*GUS* group, demonstrating effective RNAi knockdown (Figure 3a; **** *p* < 0.0001).

Subsequent to the RNAi procedure and 24 h of feeding on IVM-treated sponges, mortality rates were observed as follows: 62.38% in the dsRNA-GUS group, 60.64% in the DEPC-H_2_O group, and 40.37% in the dsRNA-*Ae-GRD* group. These findings indicate that the reduction in *Ae-GRD* expression significantly decreased the sensitivity of *Ae. aegypti* to IVM, suggesting a potential mechanism of resistance (Figure 3b; ** *p* < 0.001).

### 3.3. Changes in GABA Content after RNAi

Following the RNAi-mediated reduction in *Ae-GRD* expression, GABA content was assessed using an enzyme immunoassay to explore its impact on the *Ae-GRD* subunit and the overall GABA signaling pathway. The standard curve derived from the assay was represented by the regression equation y = 4.0221x − 0.3336 with a coefficient of determination (R^2^) of 0.9824. GABA concentrations in both experimental and control groups were calculated using this equation. The findings indicated that the alteration in *Ae-GRD* expression influenced GABA levels in *Ae. aegypti* (Figure 3c). Specifically, after dsRNA-*Ae-GRD* injection, GABA content increased by 29.57% compared with the dsRNA-*GUS* group and by 26.04% compared with the DEPC-H_2_O group. There was no significant difference observed between the two control groups.

### 3.4. Prediction of miRNAs Targeting Ae-GRD

**Figure 4:**
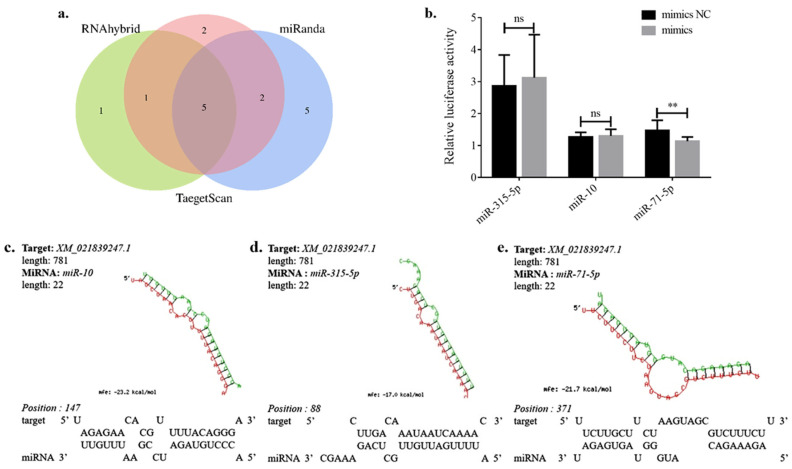
Prediction of miRNAs validated with dual-luciferase reporter assay: (**a**) Cluster analysis and Wayne plots demonstrating the results of miRNA predictions targeting *Ae-GRD*. (**b**–**d**) Binding sequences of highly scored miRNAs: miR-10, miR-315-5p, and miR-71-5p. (**e**) Results from the dual-luciferase reporter assay show that only miR-71-5p led to a significant reduction in luciferase activity by 22.93%. Data represent three biological replicates, each consisting of three technical replicates. Statistical analysis was conducted using the *t*-test. “ns” indicates no significant difference, and asterisks denote levels of statistical significance: ** *p* < 0.01.

To identify miRNAs potentially regulating the iGABAaR subunit *Ae-GRD*, three major online databases, miRanda, Targetscan, and RNAhybrid, were utilized. The predictions from these databases were analyzed by clustering and intersection (https://www.omicstudio.cn/tool/6, accessed on 9 May 2023), as illustrated in Figure 4a. For accurate target prediction, at least one region of the target mRNA’s 3′ UTR must be in base complementary pairing with positions 2–8 (the seed sequence) of the miRNA’s 5′ end [36,37]. The analysis predicted twelve miRNAs by miRanda, ten by Targetscan, and seven by RNAhybrid, with detailed predictions listed in Table S3.

Cluster analysis results suggest that five miRNAs might exert regulatory effects on *Ae-GRD*: miR-71-5p, miR-315-5p, miR-10, miR-988-5p, and miR-1890. Notably, miR-71-5p, miR-315-5p, and miR-10 emerged as the top candidates in the prediction results of all three databases. These miRNAs exhibited full pairing in the seed sequence region with the 3’ UTR of *Ae-GRD*, while showing incomplete pairing in the nonseed sequence regions (Figure 4c–e).

### 3.5. Dual-Luciferase Validation

To examine the regulatory relationship between miRNAs and their target mRNAs, dual-luciferase reporter assays were conducted. These assays verified the interaction between *Ae-GRD* and miR-71-5p, miR-315-5p, and miR-10 at the cellular level. HEK 293T cells were co-transfected with the expression reporter vector pmirGLO-*Ae-GRD* and miRNA mimics, with an NC group established for comparison. The results demonstrate that co-transfection with miR-71-5p mimics and the pmirGLO-*Ae-GRD* vector led to a 22.93% reduction in luciferase activity relative to the NC group (Figure 4b; ** *p* < 0.01). In contrast, the mimics of the other two miRNAs did not induce any significant changes in dual-luciferase activity upon co-transfection (Figure 4b). Therefore, miR-71-5p was confirmed to specifically target *Ae-GRD* in vitro.

### 3.6. miR-71-5p Regulates Ivermectin Resistance in Ae. aegypti

Previously, we demonstrated the impact of *Ae-GRD* on IVM resistance and its regulation by miR-71-5p. To further explore miR-71-5p’s role in ivermectin resistance and confirm its regulatory relationship with *Ae-GRD*, we engineered *Ae. aegypti* mosquitoes to overexpress or repress miR-71-5p via microinjection.

**Figure 5:**
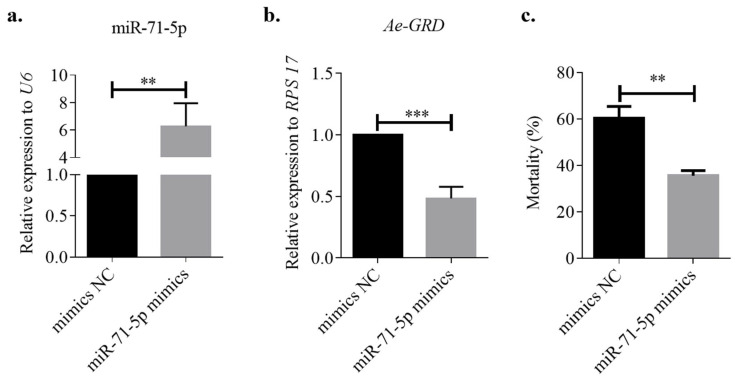
Microinjection of miR-71-5p mimics: (**a**) Injection of miR-71-5p mimics significantly increased the expression of miR-71-5p in *Ae. aegypti* compared with the control group. (**b**) Overexpression of miR-71-5p led to a substantial downregulation of *Ae-GRD* expression. (**c**) Mortality was significantly reduced in the miR-71-5p mimics group after 24 h of treatment with ivermectin. Data represent three biological replicates, each consisting of three technical replicates. Statistical analysis was conducted using the *t*-test. Asterisks denote levels of statistical significance: ** *p* < 0.01, *** *p* < 0.001.

Initially, miR-71-5p mimics were microinjected into *Ae. aegypti*, with a mimics NC group established for comparison. Assays of relative expression levels showed effective overexpression of miR-71-5p in the experimental group compared with the mimics NC group, with a 6.28-fold increase (Figure 5a; ** *p* < 0.01). This overexpression significantly repressed the transcriptional level of *Ae-GRD*, resulting in a 51.56% decrease in *Ae-GRD* expression compared with the control group (Figure 5b; *** *p* < 0.001). After feeding on an IVM-treated solution for 24 h, the mortality rate of the miR-71-5p overexpression group decreased by 34.52% compared with the control group (Figure 5c; ** *p* < 0.01).

**Figure 6:**
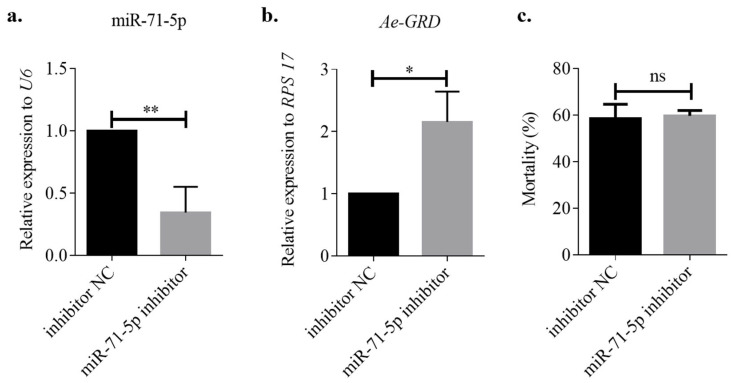
Microinjection of miR-71-5p inhibitor: (**a**) Injection of the miR-71-5p inhibitor significantly reduced the expression level of miR-71-5p by 34.40% compared with the control group. (**b**) Inhibition of miR-71-5p resulted in an upregulation of *Ae-GRD* expression. (**c**) No significant change in mortality was observed in the miR-71-5p inhibitor group after 24 h of ivermectin treatment. Data represent three biological replicates, each consisting of three technical replicates. Statistical analysis was conducted using the *t*-test. “ns” indicates no significant difference, and asterisks denote levels of statistical significance: * *p* < 0.05, ** *p* < 0.01.

Subsequently, the miR-71-5p inhibitor was microinjected to suppress miR-71-5p expression in *Ae. aegypti*, with an inhibitor NC group serving as a control. Post injection, the expression level of miR-71-5p was significantly reduced by 34.40% compared with the inhibitor NC group (Figure 6a; ** *p* < 0.01). Conversely, inhibiting miR-71-5p led to a 2.15-fold increase in *Ae-GRD* expression relative to the control group (Figure 6b; * *p* < 0.05). Interestingly, there was no significant change in mortality rates after IVM treatment compared with the control group (Figure 6c). These findings confirm that miR-71-5p directly targets *Ae-GRD* and plays a significant role in mediating IVM resistance in *Ae. aegypti* by modulating *Ae-GRD* expression.

## 4. Discussion

In this study, we identified and characterized the iGABAaR subunit *Ae-GRD* in *Ae. aegypti* for the first time. Using cDNA from female adult *Ae. aegypti* mosquitoes as a template, we cloned a partial fragment of *Ae-GRD*, and leveraging available genomic data, we obtained the full-length *Ae-GRD*. The gene and coding protein sequence of *Ae-GRD* were bioinformatically analyzed to elucidate its molecular characteristics. Sequence analysis revealed that *Ae-GRD* shares significant structural similarities with species such as the Italian honeybee, providing a theoretical basis for subsequent functional validation.

iGABAaR are established targets for insecticides such as fipronil and ivermectin [15,38]. From a molecular perspective, iGABAaR comprises both cation-permeable and anion-permeable channels [39]. Thus, in investigating the mechanisms of resistance between iGABAaR and insecticides, it is also customary to examine the types of ion channels formed by various combinations of iGABAaR subunits. iGABAaR subunits LCCH3 and GRD were first identified and studied in *Drosophila melanogaster* [12]. To date, studies on iGABAR subunits other than the RDL subunit in insects have been limited and are confined to a few species, such as Drosophila, the honeybee, and *Dictyostelium borer*. Nonetheless, existing evidence underscores the significant role of LCCH3 and GRD in neural conduction processes in insects [11,13,40].

Our observations revealed significant changes in the sensitivity of *Ae. aegypti* mosquitoes to ivermectin following RNAi treatment targeting the *Ae-GRD* subunit. Notably, adult *Ae. aegypti* exhibited a marked decrease in drug sensitivity, a finding that diverges slightly from previous studies on GRD and LCCH3 in other species [12,40]. For example, silencing RDL in *Chilo suppressalis* larvae significantly reduced their susceptibility to avermectin [40], whereas downregulation of LCCH3 and GRD mRNA levels did not impact the insecticidal activity of fluralaner [40]. Additionally, interference with RDL has been shown to alter drug susceptibility in *Ae. aegypti*, highlighting the subunit’s role in mediating responses to IVM and fluralaner [10]. In contrast, studies on the vermilion leaf mite demonstrated reduced susceptibility to abamectin and IVM following RDL interference, yet no corresponding ion channel function was detected under drug stimulation at the electrophysiological level [16]. To date, the functions of Ae-GRD and Ae-LCCH3 ion channels in *Ae. aegypti* has not been thoroughly explored, leaving the exact mechanisms by which Ae-GRD influences IVM sensitivity undetermined. Consequently, this study further investigated changes in GABA content following transcriptional interference with *Ae-GRD*, revealing that silencing of *Ae-GRD* led to a significant increase in GABA levels. This suggests that modifications in IVM sensitivity induced by interfering with *Ae-GRD* could either result from alterations in iGABA receptor-gated ion channel functions or adjustments within other components of the GABAergic neural pathway. The precise mechanisms require further elucidation through comprehensive electrophysiological studies and other experimental approaches.

Furthermore, miRNAs regulate gene expression by binding to the mRNAs of target genes, leading to mRNA degradation or inhibition of translation processes [41]. Establishing the relationship between miRNAs and target genes involves extensive predictive screening and experimental validation of these predictions. In this study, we obtained the complete sequence of the 3’ UTR region of *Ae-GRD* from the NCBI database. Target prediction was performed using TargetScan, miRanda, and RNAhybrid, yielding high-scoring cross-predictions for miR-71-5p, miR-315-5p, and miR-10. The dual-luciferase reporter assay confirmed that miR-71-5p inhibited luciferase activity, indicating regulation of *Ae-GRD* by miR-71-5p at the cellular level.

Further validation involved overexpressing and repressing miR-71-5p in *Ae. aegypti* through microinjection. Overexpression of miR-71-5p significantly suppressed *Ae-GRD* transcription, while repression of miR-71-5p markedly increased it. The involvement of miRNAs in various physiological and functional processes of insect growth and development is well documented, though reports on miR-71-5p in *Ae. aegypti* are scarce. Building on findings from other species, we explored whether miR-71-5p’s regulation of *Ae-GRD* expression contributes to IVM resistance development in *Ae. aegypti* through bioassays. After manipulating miR-71-5p expression in adult *Ae. aegypti*, both miRNA and target gene expression levels were analyzed. Overexpression of miR-71-5p significantly reduced *Ae-GRD* expression, correlating with decreased mortality rates following IVM treatment. miRNAs are integral to physiological activities in insects by regulating target gene expression. For instance, in the silkworm, miR-281 regulates molting by affecting the ecdysteroid receptor [42]; in the Italian honeybee, miR-184-3p, miR-276-3p, miR-87-3p, and miR-124-3p are significantly associated with olfactory learning and memory [41]. Moreover, miR-2, miR-13a, miR-13b, and miR-71 regulate egg formation in migratory locusts [43], while miR-71 and miR-263 co-regulate chitin synthase and chitinase to control molting in locusts [44]; miR-223 offers neuroprotective effects by targeting glutamate receptors [45].

## 5. Conclusions

In this study, we successfully identified and characterized the iGABAR subunit *Ae-GRD* in *Ae. aegypti* for the first time, revealing that it is highly homologous and well-conserved across various species. *Ae-GRD* is a target gene of miR-71-5p, and there exists a negative transcriptional correlation between them. The downregulation of *Ae-GRD* expression leads to a decreased sensitivity of *Ae. aegypti* to IVM, suggesting a potential mechanism for resistance. Furthermore, miR-71-5p appears to play a role in the development of IVM resistance in *Ae. aegypti* by regulating the expression of *Ae-GRD*. These findings provide insights into the molecular interactions affecting insecticide resistance, offering potential targets for controlling this vector species more effectively.

## Data Availability

The data presented in this study are available in the main text of the article.

## Supplementary Materials

The following supporting information can be downloaded at: https://www.mdpi.com/article/10.3390/insects15060453/s1: Table S1: The primers used in this experiment; Table S2: Sequence information of GRD in different species of insects; Table S3: Prediction of miRNAs targeting *Ae-GRD*.
