# Supplementary material for: Impact of Ae-GRD on Ivermectin Resistance and Its Regulation by miR-71-5p in Aedes aegypti
Source: Insects. 2024 Jun 14;15(6):453. doi: 10.3390/insects15060453 (PMC11203581; doi:10.3390/insects15060453)
Supplement: Supplementary file 1 [file insects-15-00453-s001.zip › insects-3031229-supplementary.pdf]

Table S1. The primers used in this experiment

Primer name	Primer sequences (5′-3′)	Usage
Ae-GRD-1F	CTCTAAAGGCGGAATCACC	cDNA cloning
Ae-GRD-1R	CTTTCCCGACAACGATCC	
T7-dsGRD-F1	taatacgactcactatagggACATCTACAACACAGCCCCG	RNA interference
T7-dsGRD-R1	TGCATCAGACAGGACGCTTT	
T7-dsGRD-F2	ACATCTACAACACAGCCCCG	
T7-dsGRD-R2	taatacgactcactatagggTGCATCAGACAGGACGCTTT	
T7-dsGUS-F1	taatacgactcactatagggGCGGCCGCCCCTTACGCTGAAGAGATGC	
T7-dsGUS-R1	CTCGAGGGCACAGCACATCAAAGAGA	
T7-dsGUS-F2	GCGGCCGCCCCTTACGCTGAAGAGATGC	
T7-dsGUS-R2	taatacgactcactatagggCTCGAGGGCACAGCACATCAAAGAGA	
miR-315-5p mimics-F	UUUUGAUUGUUGCUCAGAAAGC	Synthesis of microRNA mimics and NC
miR-315-5p mimics-R	UUUCUGAGCAACAAUCAAAAUU	
miR-10 mimics-F	ACCCUGUAGAUCCGAAUUUGUUTT	
miR-10 mimics-R	AACAAAUUCGGAUCUACAGGGUTT	
miR-71-5p mimics-F	AGAAAGACAUGGGUAGUGAGAUTT	
miR-71-5p mimics-R	AUCUCACUACCCAUGUCUUUCUTT	
mimics NC-F	UUCUCCGAACGUGUCACGUTT	
mimics NC-R	ACGUGACACGUUCGGAGAATT	
miR-71-5p inhibitor	AUCUCACUACCCAUGUCUUUCU	Synthesis of microRNA inhibitor and NC
inhibitor NC	CAGUACUUUUGUGUAGUACAA	

Table S1. The primers used in this experiment (continued)

**Primer name**	**Primer sequences (5′-3′)**	**Usage**
miR-71-5p	GTCGTATCCAGTGCAGGGTCCGAGGTATT CGCACTGGATACGACATCTCA	RT stem-loop primer
U6	GTCGTATCCAGTGCAGGGTCCGAGGTATT CGCACTGGATACGACAAAAAT	
Ae-GRD-qF	AGGTGTACGTTCCGTGCATT	qPCR
Ae-GRD-qR	AGATCCGTCCTCGAGTCCAA	
RPS17-qF	AAGAAGTGGCCATCATTCCA	
RPS17-qR	GGTCTCCGGGTCGACTTC	
miR-71-5p-qF	CGCGAGAAAGACATGGGTAG	
miR-71-5p-qR	AGTGCAGGGTCCGAGGTATT	
U6-qF	CGCAAGGATGACACGCAAA	
U6-qR	GTCGTATCCAGTGCAGGG	

Table S2. Sequence information of GRD in different species of insect

Species	Protein sequence numbers	Length (aa)
Aedes aegypti	XP_021694939.1	683
Aedes albopictus	XP_062700266.1	688
Armigeres subalbatus	XP_062539564.1	674
Culex pipiens pallens	XP_039440121.1	702
Culex quinquefasciatus	XP_038105041.1	701
Toxorhynchites rutilus septentrionalis	XP_055618950.1	700
Wyeomyia smithii	XP_055522907.1	699
Anopheles arabiensis	XP_040174363.1	736
Anopheles sinensis	KFB34891.1	630
Anopheles maculipalpis	XP_050081393.1	732
Anopheles gambiae	XP_001237369.3	736
Anopheles merus	XP_041764767.1	734
Anopheles nili	XP_053678580.1	739
Anopheles funestus	XP_049286866.1	748
Drosophila melanogaster	NP_001162770.1	510
Hermetia illucens	XP_037920157.1	609
Ceratitis capitata	XP_012161002.1	739
Bactrocera latifrons	XP_018796579.1	735
Anastrepha ludens	XP_053949317.1	734
Pseudolycoriella hygida	KAJ6638698.1	582
Glossina fuscipes	XP_037894652.1	485
Musca domestica	XP_019893131.1	589
Psylliodes chrysocephala	CAH1106177.1	481
Tribolium castaneum	EFA02907.2	557
Helicoverpa armigera	PZC75662.1	576
Spodoptera frugiperda	XP_035431640.1	564
Vespa mandarinia	XP_035726979.1	536

Table S2. Sequence information of GRD in different species of insect (continued)

Gene Name	Gene ID	Length (aa)
Apis mellifera caucasica	KAG6804607.1	509
Apis mellifera	XP_026298420.1	520
Nasonia vitripennis	XP_008203400.1	569
Bombyx mori	NP_001182633.1	594
Ixodes scapularis	XP_040062219.1	525
Tetranychus urticae	AGC31413.1	

P.S: aa (amino acids).

Table S3. Prediction of miRNAs targeting *Ae-GRD*

Gain Way	Name	Sequences (5′-3′)
miRanda	miR-71-5p	AGAAAGACAUGGGUAGUGAGAU
	miR-11923	ACAACGGCAGCCGGAACGAUCU
	miR-315-5p	UUUUGAUUGUUGCUCAGAAAGC
	miR-10	ACCCUGUAGAUCCGAAUUUGUU
	miR-11917	CUGAAAACUUAAUCGAUUGGUC
	miR-263a-5p	AAUGGCACUGGAAGAAUUCACGG
	miR-315-3p	CUUUCGAGCAGUAAUCAAAGUC
	miR-375	UUUGUUCGUUUGGCUCGAGUUA
	miR-11925	CUGUCGAGCCGGUUGAACCAC
	miR-988-5p	GUGUGCUUUGUGACAAUGAGA
	miR-1890	UGAAAUCUUUGAUUAGGUCUGG
TargetScan	miR-79-3p	UAAAGCUAGAUUACCAAAGCAU
	miR-71-5p	AGAAAGACAUGGGUAGUGAGAU
	miR-315-5p	UUUUGAUUGUUGCUCAGAAAGC
	miR-10	ACCCUGUAGAUCCGAAUUUGUU
	bantam-5p	CCGGUUUUCAUUUUCGAUCUGAC
	miR-375	UUUGUUCGUUUGGCUCGAGUUA
	miR-988-5p	GUGUGCUUUGUGACAAUGAGA
	miR-1890	UGAAAUCUUUGAUUAGGUCUGG
	miR-79-3p	UAAAGCUAGAUUACCAAAGCAU
	miR-11-5p	AGAACUCCGGCUGUGACCUGUG
	miR-210	CUUGUGCGUGUGACAACGG

Table S3. Prediction of miRNAs targeting *Ae-GRD* (continued)

Gain Way	Name	Sequences (5′-3′)
RNAhybrid	miR-315-5p	UUUUGAUUGUUGCUCAGAAAGC
	miR-10	ACCCUGUAGAUCCGAAUUUGUU
	miR-71-5p	AGAAAGACAUGGGUAGUGAGAU
	miR-988-5p	GUGUGCUUUGUGACAAUGAGA
	bantam-5p	CCGGUUUUCAUUUUCGAUCUGAC
	miR-1890	UGAAAUCUUUGAUUAGGUCUGG
	miR-11900	AUUUUUUUGACUGUAAUUUUAUAG

P.S: The results obtained by the three tools are highlighted in red font after they are crossed.
